# Ionomic Parameters of Populations of Common Juniper (*Juniperus communis* L.) Depending on the Habitat Type

**DOI:** 10.3390/plants12040961

**Published:** 2023-02-20

**Authors:** Lina Jocienė, Edvina Krokaitė, Tomas Rekašius, Ramūnas Vilčinskas, Asta Judžentienė, Vitas Marozas, Eugenija Kupčinskienė

**Affiliations:** 1 Department of Biology, Faculty of Natural Sciences, Vytautas Magnus University, K. Donelaičio Str. 58, 44248 Kaunas, Lithuania; 2 Department of Mathematics and Statistics, Faculty of Informatics, Vytautas Magnus University, K. Donelaičio Str. 58, 44248 Kaunas, Lithuania; 3 Department of Mathematical Statistics, Faculty of Fundamental Sciences, Vilnius Gediminas Technical University, Saulėtekio Ave. 11, 10223 Vilnius, Lithuania; 4 Department of Organic Chemistry, Center for Physical Sciences and Technology, Saulėtekio Ave. 3, 10257 Vilnius, Lithuania; 5 Institute of Biosciences, Life Sciences Center, Vilnius University, Saulėtekio Ave. 7, 10257 Vilnius, Lithuania; 6 Department of Environment and Ecology, Faculty of Forest Sciences and Ecology, Vytautas Magnus University, K. Donelaičio Str. 58, 44248 Kaunas, Lithuania

**Keywords:** nutrition, nutrients, macroelements, microelements, non-essential elements, heavy metals, needles, Ellenberg indicatory values, EIV, DNA polymorphism

## Abstract

For the study of the ionomic parameters of *Juniperus communis* needles, fourteen sites covering most of the territory of Lithuania and belonging to distinct habitats (coastal brown dunes covered with natural Scots pine forests (G), *Juniperus communis* scrubs (F), transition mires and quaking bogs (D), subcontinental moss Scots pine forests (G), and xero-thermophile fringes) were selected. Concentrations of macro-, micro-, and non-essential elements were analyzed in current-year needles, sampled in September. According to the concentrations of elements in *J. communis* needles, the differences between the most contrasting populations were as follows: up to 2-fold for Mg, N, K, Ca, and Zn; 2- to 7-fold for P, Na, Fe, Cu, Al, Cr, Ni, and Pb; and 26- to 31-fold for Mn and Cd. The concentrations of Cd, Cr, and Ni in needles of *J. communis* did not reach levels harmful for conifers. When compared to all other habitats (B, F, G, and E), the populations from transition mires and quaking bogs (D) had significantly lower concentrations of main nutritional elements N (12176 µg/g d. m.), P (1054 µg/g d. m.), and K (2916 µg/g d. m.). In *Juniperus communis* scrubs (F), a habitat protected by EUNIS, the concentration of K in the needles was highest, while Zn and Cu concentrations were the lowest. Principal component (PC) analyses using concentrations of 15 elements as variables for the discrimination of populations or habitats allowed authors to distinguish F and B habitats from the E habitat (PC1) and F and D habitats from the G habitat (PC2). Discriminating between populations, the most important variables were concentrations of P, N, Mg, Ca, Cu, and K. Discriminating between habitats, the important variables were concentrations of N and P.

## 1. Introduction

Common juniper (*Juniperus communis* L.) is a coniferous tree or shrub belonging to the Cupressaceae family. It is one of the most widespread plant species in the world [1]. *Juniperus communis* grows in the northern hemisphere [2]. Its distribution range encompasses four continents, with a European range from Ireland to Russia in longitude and from Spain to Scandinavia in latitude [3]. Although *J. communis* is the most widespread conifer in the world, it has become an endangered species in some European locations [4]. An increased threat of juniper’s extinction is described in Ireland [5], Britain [6,7], Belgium [8,9,10,11], The Netherlands [12], Germany [13,14], and Czech Republic [15].

*Juniperus communis* is one of the three conifer species that grow naturally in the Baltic countries [16,17,18,19]. It is one (ssp. *communis* (Syme)) of four juniper’s subspecies (ssp. *communis* (Syme), ssp. *nana* (Hook), ssp. *hemisphaerica* (J. and C. Presl) Nyman, ssp. *depressa* (Pursh) Franko) [3]. In Lithuania *J. communis* is the most abundant in *Pinus sylvestris* stands aged 60–120 years [16]. As with neighboring countries, the viability of *J. communis* is declining in Lithuania [16,17,18,19], likely because the number of its individuals is decreasing: the abundance of *J. communis* decreased by 1.5 times between 2002 and 2017 [16]. One of the reasons might be the aging of junipers. With aging, especially beyond 70 years, *J. communis* reproduction decreases [6]. Climate change, fragmentation, diseases, and increasing tourism are posing additional threats [17,18]. In addition, the damage caused by ungulates increased 9.1 times between 2002 and 2017 [16]. *Juniperus communis* is highly sensitive to surface fires: juniper was absent in fire-affected 70 years old stands of *P. sylvestris* in South Lithuania, though it was an abundant species in areas without fire events [18]. *Juniperus communis* is a very ornamental species providing special value to the landscape [3], and its stands are a beloved subject for recreation in Baltic countries, especially in Lithuania [16,17]. Compounds extracted from *J. communis* have been assessed using model species or tissue cultures for potential use in veterinary care and medicine, e.g., effects on Freund’s adjuvant-induced arthritis in rats [20], melanogenesis on zebrafish pigmentation [21], in human SH-SY5Y neuroblastoma cells in relation to diabetes [22], and showing in vitro antioxidant, genotoxic and cytotoxic activities [23]. *Juniperus communis* needles, seeds (usually titled as berries), and bark are used in veterinary and folk medicine for the treatment of respiratory diseases [24,25,26], diabetes [22], and inflammatory diseases [27], and also as antibacterial and antifungal agents [28,29]. Due to its important ecological and economic role [30,31], there is a significant interest in the biology of *J. communis* in Baltic countries [32,33,34].

*Juniperus communis* is a slow growing coniferous tree or shrub [1,2,3]. The common juniper, as a species of extremely wide geographical distribution, is adapted to various environmental factors. It is found in various habitats and in elevations up to 2400 m. The species grows in a variety of soils and is found in open grasslands in pure or mixed stands. In Lithuania, *J. communis* thrives in pine forests on dry poor soils, but also occurs in mixed forests, peatlands, or near water [16,17,18]. The common juniper is tolerant to drought and low temperatures [2]. It primarily grows as a shrub and is light demanding, thriving in open sites and suffering from shade caused by dense tree cover. These environmental features of *J. communis* correspond to numeric characteristics (in the scale of 1 to 9) defined by Ellenberg et al. [35], where soil richness (N) is rated as wide range—(x), as well as wide range soil acidity (x), below average soil moisture (4), and high light intensity (8). In Europe, about forty habitats of *J. communis* are distinguished [36,37]; this number is lower in the Baltic countries. The predominant type of *J. communis* habitat in Lithuania is the subcontinental mossy Scots pine forests that are common throughout the country [16]. The second most common juniper habitat is xero-thermophilic fringes, primarily located in the central and southern parts of Lithuania [30]. *Juniperus communis* scrubs (representing the places protected by EUNIS [36]) and coastal brown dunes covered with naturally growing Scots pine are very small areas in Lithuania. Areas of transitional mires and quaking bogs are also rare in the country.

For more accurate usage of the species, comprehensive knowledge of *J. communis* condition is needed. The health of the species might be related to the extent of genetic diversity [1,38,39] and nutritional state in terms of elements’ concentrations. Several studies in multiple European countries have indicated that the fertility, humidity, and temperature of the soil under the shrubs of *J. communis* is different when compared to open areas [40,41,42]. The presence of *J. communis* caused an increase in the amount of C, N, P and Ca^2+^, Mg^2+^, K^+^, and Na^+^ in the upper layer of the soil when compared to open areas. Comparison of elements in the precipitation collected in open areas and under *J. communis* (a Lithuanian case study) revealed higher amounts of Mn, Fe, and Zn, a slight increase in Ni, Cr, and Cd, and a decrease in Pb [40] under the shrubs.

Although ionome investigations have been conducted for various species of *Juniperus* [35,36,37,38,39,40,41,42,43,44,45], including *J. communis* [40,46], too little attention has been paid to ionome interactions with environment. The present study evaluated the ionomic parameters of *J. communis* populations in relation to habitat type.

## 2. Results

### Concentration of Macro-, Micro-, and Non-Essential Elements in Current-Year Needles of J. communis

**Figure 1:**
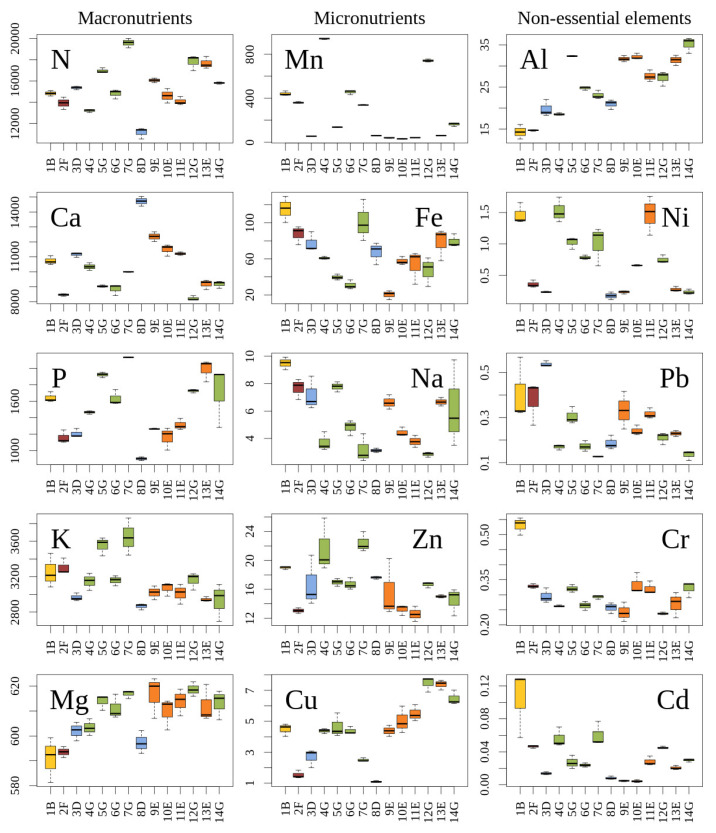
Concentrations (µg/g d. m. in y axes) of macroelements (N, Ca, P, K, Mg), microelements (Mn, Fe, Na, Zn, Cu) and non-essential elements (Al, Ni, Pb, Cr, Cd) in current-year needles of Lithuanian populations of *J. communis*; x axes—titles of populations (title of each population consists of 2 characters: a number denotes geographical location (see map in Materials and Methods section), a letter denotes the type of habitat, boxes of different color denote distinct habitats). The central line of each box indicates a median value; the boxes, the lower (25%) and upper (75%) quartiles, and the whiskers are from 10 to 90 percentiles (typical range).

The concentrations of macroelements in the needles of *J. communis* populations ranged from 11147 to 19532 μg/g d. m. for N, from 903 to 2133 μg/g d. m. for P, from 2865 to 3648 μg/g d. m. for K (N, P, and K concentrations were lowest in the population 8 D, and highest in the population 7 G), from 591 to 619 μg/g d. m. for Mg (with the lowest concentration in the 1 B and the highest concentration in the 12 G), and from 8224 to 14723 μg/g d. m. for Ca (with the lowest concentration in the 12 G, and the highest concentration in the 8 D) (Figure 1: location of populations is provided in the map in Materials and Methods section).

The concentrations of microelements (Mn, Fe, Na, Zn, Cu) in the needles of *J. communis* populations ranged from 30.2 to 939.2 μg/g d. m. for Mn (with lowest concentration in the population 10 E, and the highest concentration in the 4 G), from 20.2 to 115 μg/g d. m. for Fe (with the lowest concentration in the 9 E, and the highest in the 1 B), from 2.84 to 9.49 μg/g d. m. for Na (with the lowest concentration in the 12 G, and the highest in the 1 B), from 12.58 to 22.42 μg/g d. m. for Zn (with the lowest concentration in the 11 E, and the highest in the population 7 G), and from 1.10 to 7.46 μg/g d. m. for Cu (with the lowest concentration in the 8 D, the highest in the 12 G).

The concentrations of non-essential elements (Al, Ni, Pb, Cr, Cd) in the needles of *J. communis* ranged from 14.35 to 35.15 μg/g d. m. for Al (with the lowest concentration in the 14 G and the highest concentration in the 1 B), from 0.126 to 0.537 μg/g d. m. for Pb (with the lowest concentration in the 7 G and the highest concentration in the 3 D), from 0.18 to 1.52 μg/g d. m. for Ni (with the lowest concentration in the 8 D, and the highest concentration in the 4 G), from 0.242 to 0.531 μg/g d. m. for Cr (with the lowest concentration in the 9 E and the highest concentration in the 1 B), and from 0.004 to 0.105 μg/g d. m. for Cd (with the lowest concentration in the 10 E and the highest concentration in the 1 B).

Differences between the most contrasting populations in terms of elemental concentrations in current-year needles of *J. communis* were as follows: N—1.76 times; P—2.36 times; K—1.27 times; Mg—1.05 times; Ca—1.79 times; Na—3.34 times; Fe—5.71 times; Mn—31.1 times; Zn—1.79 times; Cu—6.78 times; Al—2.45 times; Cr—2.23 times; Ni—6.61 times; Pb—4.26 times; Cd—26.35 times.

**Figure 2:**
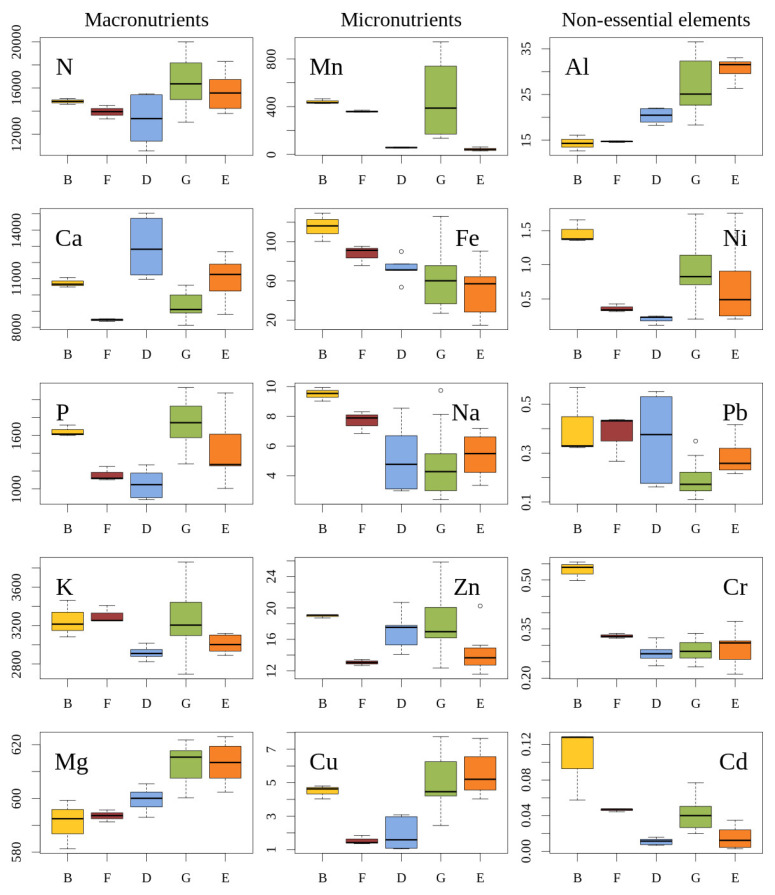
Concentrations (µg/g d. m., y axes) of macroelements (N, Ca, P, K, Mg), microelements (Mn, Fe, Na, Zn, Cu), and non-essential elements (Al, Ni, Pb, Cr, Cd) in current-year needles of *J. communis* from different habitats. X axes—types of habitats: B—coastal brown dunes covered with natural Scots pine forests, F—*Juniperus communis* scrubs, D—transition mires and quaking bogs, G—subcontinental moss Scots pine forests, E—xero-thermophile fringes. The central line of each box indicates a median value; the boxes, the lower (25%) and upper (75%) quartiles, and the whiskers are from 10 to 90 percentiles (typical range), the points are outliers.

The comparison of populations according to habitat type (Figure 2) showed that the concentration of N in the needles of *J. communis* were 1.14 to 1.37 times lower (*p* < 0.05) in the transition mires and quaking bogs (D) when compared to other habitats (coastal brown dunes covered with natural Scots pine forests (G), *Juniperus communis* scrubs (F), subcontinental moss Scots pine forests (G), and xero-thermophile fringes (E)). Needle concentration of N was 1.20 to 1.37 times higher (*p* < 0.05) in the subcontinental moss Scots pine forests (G) compared to the coastal brown dunes covered with natural Scots pine forests (B), *J. communis* scrubs (F), and transition mires and quaking bogs (D).

The concentration of Ca in the needles of *J. communis* was 1.27 to 1.31 times lower (*p* < 0.05) in *Juniperus communis* scrubs (F) than in coastal brown dunes covered with natural Scots pine forests (B) and transition mires and quaking bogs (D). In the transition mires and quaking bogs (D), Ca concentration was 1.20 to 1.53 times higher (*p* < 0.05) when compared to coastal brown dunes covered with natural Scots pine forests (B), *Juniperus communis* scrubs (F), and subcontinental moss Scots pine forests (G). In coastal brown dunes covered with natural Scots pine forests (B), the concentration of Ca was 1.20 to 1.27 times higher (*p* < 0.05) than that in *Juniperus communis* scrubs (F) and subcontinental moss Scots pine forests (G). In xero-thermophile fringes (E), the concentration of Ca was 1.19 to 1.31 times higher (*p* < 0.05) than that in *Juniperus communis* scrubs (F) and subcontinental moss Scots pine forests (G). The concentration of P in the needles of *J. communis* was 1.42 to 1.56 times lower (*p* < 0.05) in subcontinental moss Scots pine forests (G) when compared to *Juniperus communis* scrubs (F) and transition mires and quaking bogs (D), 1.52 to 1.67 times higher (*p* < 0.05) in subcontinental moss Scots pine forests (G) than in *Juniperus communis* scrubs (F) and transition mires and quaking bogs (D), and 1.36 times (*p* < 0.05) higher in xero-thermophile fringes (E) when compared to transition mires and quaking bogs (D). The concentration of K in the needles of *J. communis* was 1.12 to 1.13 times lower (*p* < 0.05) in transition mires and quaking bogs (D) than in coastal brown dunes covered with natural Scots pine forests (B), *Juniperus communis* scrubs (F), and subcontinental moss Scots pine forests (G), and 1.08 times lower (*p* < 0.05) in xero-thermophile fringes (E) than in *Juniperus communis* scrubs (F). The concentration of Mg in the needles of *J. communis* was 1.02 to 1.04 times higher (*p* < 0.05) in subcontinental moss Scots pine forests (G) when compared to coastal brown dunes covered with natural Scots pine forests (B), *Juniperus communis* scrubs (F), and transition mires and quaking bogs (D).

The concentration of Mn in the needles of *J. communis* was 1.23 to 10.45 times higher (*p* < 0.05) in coastal brown dunes covered with natural Scots pine forests (B) when compared to *J. communis* scrubs (F), transition mires and quaking bogs (D), and xero-thermophile fringes (E). In *Juniperus communis* scrubs (F) and subcontinental moss Scots pine forests (G), the concentration of Mn was 6.35 to 8.53 and 8.14 to 10.94 times higher (*p* < 0.05), respectively, when compared to transition mires and quaking bogs (D) and xero-thermophile fringes (E). The concentration of Fe in the needles of *J. communis* was 1.59 to 2.20 times higher (*p* < 0.05) in coastal brown dunes covered with natural Scots pine forests (B) than in transition mires and quaking bogs (D), subcontinental moss Scots pine forests (G), and xero-thermophile fringes (E). For the *Juniperus communis* scrubs (F) the concentration of Fe was 1.46 to 1.67 times higher (*p* < 0.05) when compared to subcontinental moss Scots pine forests (G) and xero-thermophile fringes (E). The concentration of Na in the needles *of J. communis* was 1.24 to 2.00 times higher (*p* < 0.05) in coastal brown dunes covered with natural Scots pine forests (B) when compared to other habitats. For *Juniperus communis* scrubs (F), the concentration of Na was 1.43 to 1.61 times higher (*p* < 0.05) than that in subcontinental moss Scots pine forests (G) and xero-thermophile fringes (E). The concentration of Zn in the needles of *J. communis* was 1.45 to 1.31 times higher (*p* < 0.05) in *Juniperus communis* scrubs (F) when compared to xero-thermophile fringes (E). In subcontinental moss Scots pine forests (G) the concentration of Zn was 1.29 times higher (*p* < 0.05) than in xero-thermophile fringes (E). For the *Juniperus communis* scrubs (F), the concentration of Zn was 1.31 to 1.39 times lower (*p* < 0.05) when compared to that in transition mires and quaking bogs (D) and subcontinental moss Scots pine forests (G). The concentration of Cu in the needles of *J. communis* was 2.37 to 2.91 times higher (*p* < 0.05) in coastal brown dunes covered with natural Scots pine forests (B) when compared to *Juniperus communis* scrubs (F) and transition mires and quaking bogs (D). In subcontinental moss Scots pine forests (G) and xero-thermophile fringes (E) the concentration of Cu was 2.63 to 3.23 and 2.94 to 3.62 times higher (*p* < 0.05), respectively, than that in transition mires and quaking bogs (D) and xero-thermophile fringes (E).

The concentration of Al in the needles of *J. communis* was 1.42 to 2.14 times lower (*p* < 0.05) in coastal brown dunes covered with natural Scots pine forests (B) when compared to other habitats. For *Juniperus communis* scrubs (F), the concentration of Al was 1.39 to 2.09 times lower (*p* < 0.05) when compared to other habitats. In transition mires and quaking bogs (D), the concentration was 1.32 to 1.51 times lower (*p* < 0.05) than that in subcontinental moss Scots pine forests (G) and xero-thermophile fringes (E). The concentration of Ni in the needles of *J. communis* was 1.65 to 7.10 times higher (*p* < 0.05) in coastal brown dunes covered with natural Scots pine forests (B) when compared to other habitats. In subcontinental moss Scots pine forests (G), the concentration of Ni was 2.47 higher (*p* < 0.05) than in *Juniperus communis* scrubs (F). In transition mires and quaking bogs (D), the concentration of Ni was 3.20 to 4.30 times lower (*p* < 0.05) than in coastal brown dunes covered with natural Scots pine forests (B), *Juniperus communis* scrubs (F), subcontinental moss Scots pine forests (G), and xero-thermophile fringes (E). The concentration of Pb in the needles of *J. communis* was 1.50 to 2.18 times lower (*p* < 0.05) in subcontinental moss Scots pine forests (G) when compared to *Juniperus communis* scrubs (F) and xero-thermophile fringes (E). The concentration of Cr in the needles of *J. communis* was 1.19 to 1.92 times higher (*p* < 0.05) in coastal brown dunes covered with natural Scots pine forests (B) when compared to other habitats. For *Juniperus communis* scrubs (F), the concentration of Cr was 1.16 to 1.19 times higher (*p* < 0.05) than in transition mires and quaking bogs (D) and subcontinental moss Scots pine forests (G). The concentration of Cd in the needles of *J. communis* was 2.26 to 9.51 times higher (*p* < 0.05) in coastal brown dunes covered with natural Scots pine forests (B) when compared to other habitats. For *Juniperus communis* scrubs (F), the concentration of Cd was 3.21 to 3.67 times higher (*p* < 0.05) when compared to that in transition mires and quaking bogs (D) and xero-thermophile fringes (E). In subcontinental moss Scots pine forests (G), the concentration was 2.80 to 3.67 times higher (*p* < 0.05) than that in transition mires and quaking bogs (D) and xero-thermophile fringes (E).

**Figure 3:**
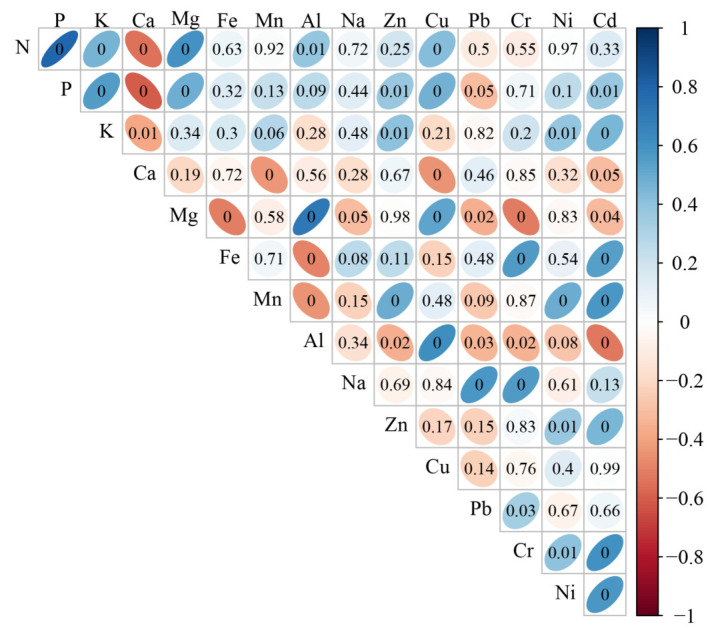
The correlogram (Pearson rank correlation coefficients Rs from −1 to 1) for all pairs of elements examined for *J. communis* populations. Blue color represents positive and red color represents negative correlations. Color intensity indicates the strength of correlation, so the stronger the correlation, the darker the color. Correlation numbers inside colored figures indicate the level of significance: thin ellipsoid figures show significant (*p* < 0.05) correlations, while figures towards spherical shape showinsignificant correlations. N, Ca, P, K, and Mg (macroelements), Mn, Fe, Na, Zn, and Cu (microelements), Al, Ni, Pb, Cr, and Cd (non-essential elements).

To relate concentrations of macro-, micro-, and non-essential elements of *J. communis* populations, Pearson rank correlations were estimated (Figure 3). The concentration of N was positively correlated with the concentrations of P (Rs = 0.79, *p* < 0.001) and Mg (Rs = 0.60, *p* < 0.0054) and negatively correlated with Ca (Rs = –0.54, *p* < 0.020). The concentration of P was positively correlated with the concentration of K (Rs = 0.55, *p* < 0.015) and negatively correlated with Ca (Rs = –0.60, *p* < 0.002). The concentration of Mg was positively correlated with the concentration of Al (Rs = 0.71, *p* < 0.001) and Cu (Rs = 0.53, *p* < 0.032) and negatively with Cr (Rs = –0.52, *p* < 0.042). The concentration of Fe was positively correlated with concentrations of Cr (Rs = 0.57, *p* < 0.009) and Cd (Rs = 0.55, *p* < 0.016). The concentration of Mn was positively correlated with the concentration of Cd (Rs = 0.57, *p* < 0.007). The concentration of Al was positively correlated with the concentration of Cu (Rs = 0.62, *p* < 0.01) and negatively correlated with Cd (Rs = –0.52, *p* < 0.035). The concentration of Na was positively correlated with the concentration of Pb (Rs = 0.57, *p* < 0.007) and Cr (Rs = 0.56, *p* < 0.010). The concentration of Cr was positively correlated with the concentration of Cd (Rs = 0.60, *p* < 0.003). The concentration of Ni was positively correlated with the concentration of Cd (Rs = 0.58, *p* < 0.006). Concentration of K, Ca, Zn, Cu, and Pb did not correlate with concentration of any other element.

**Figure 4:**
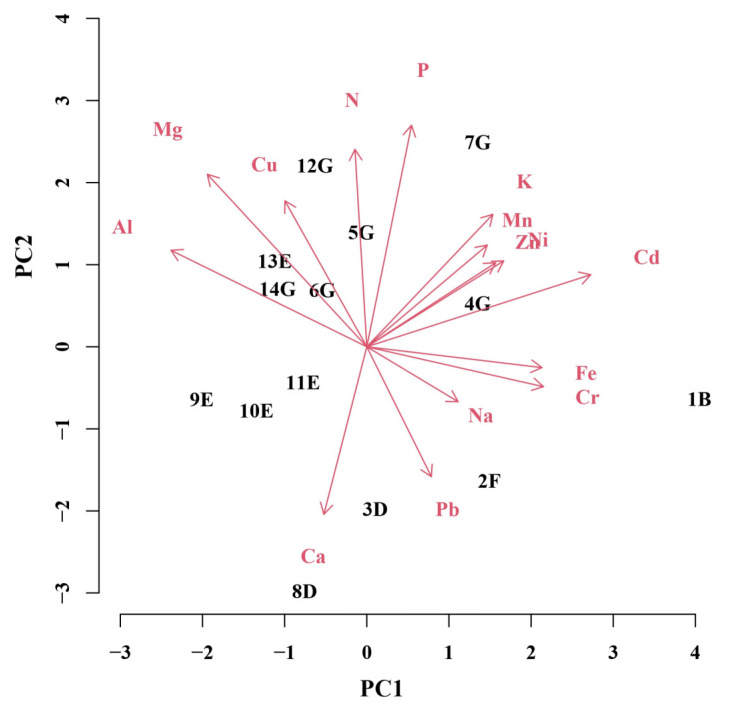
Principal component analysis for the first two principal components of a model testing variation of *J. communis* populations depending on concentrations of macroelements (N, Ca, P, K, and Mg), microelements (Mn, Fe, Na, Zn, and Cu) and non-essential elements (Al, Ni, Pb, Cr, and Cd) as variables (red arrows with letters). Title of each population consists of 2 characters: a number denotes geographical location (see the map in Materials and Methods section) and a letter denotes the type of a habitat.

To explain the most important macroelements, microelements, and non-essential elements on the variability of *J. communis* populations, principal component (PC) analysis was performed (Figure 4). The first four PCs were highly informative and together accounted for 79.8% of the overall variance. Individually, PC1, PC2, PC3, and PC4 accounted for 29.2% (4.071), 27.1% (3.846), 15.2% (2.103), and 8.3% (1.262) of variance, respectively. Contribution of most elements to PC1 was positive and that of N, Ca, Cu, Mg, and Al was negative. The contribution of most elements to PC2 was positive, and it was negative in case of Fe, Cr, Na, Pb, and Ca. According to the variability of elemental concentrations, displayed in the biplot of two PC components, the importance of variables (in descending order) in PC1 was as follows: Cd > Al > Cr > Fe > Mg > Ni > Zn > K > Mn > Na > Cu > Pb > P > Ca > N. The order of importance of variables in PC2 was different: P > N > Mg > Ca > Cu > K > Pb > Mn > Al > Ni > Zn > Cd > Na > Cr > Fe.

**Figure 5:**
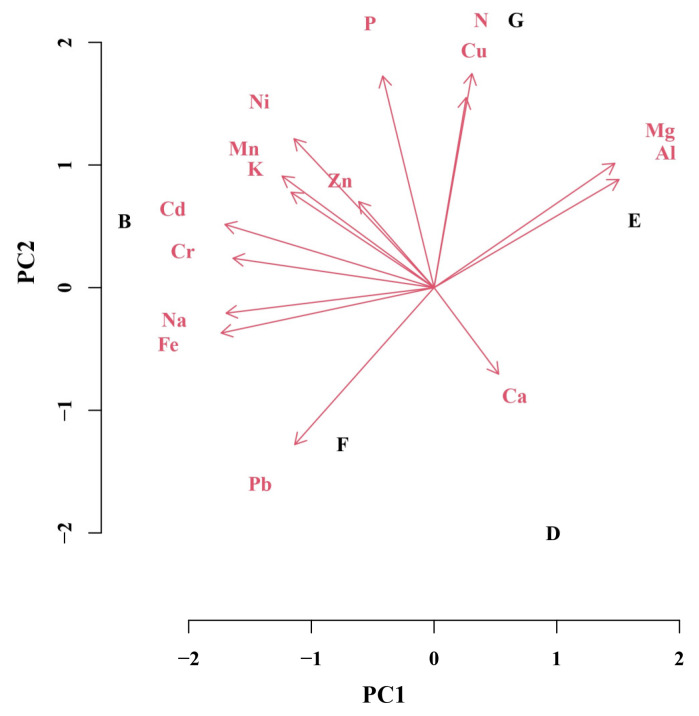
Principal component analysis for the first two principal components of a model testing variation of *J. communis* habitats depending on the concentration of macroelements (N, Ca, P, K, and Mg), microelements (Mn, Fe, Na, Zn, and Cu), and non-essential elements (Al, Ni, Pb, Cr, and Cd) as variables (red arrows with letters). Black letters denote habitat type: B—coastal brown dunes covered with natural Scots pine forests, F—*Juniperus communis* scrubs, D—transition mires and quaking bogs, G—subcontinental moss Scots pine forests, and E—xero-thermophile fringes.

PCA analyses of populations by habitat type (Figure 5) showed that the first four PCs accounted for all (100%) of the variance. Individually, PC1, PC2, PC3, and PC4 accounted for 46.6% (4.071), 33.9% (3.846), 14.0% (2.103), and 5.5% (1.262) of variance, respectively. Contribution of Cu, N, Ca, Mg, and Al to PC1 was positive, while it was negative in case of P, Ca, Cu, Mg, Pb, Ni, Mn, K, Zn, Cd, Fe, Cr, and Na. For variability of PC2, contributions of most elements were positive, while contributions of Na, Fe, Ca, and Pb were negative. According to the variability of elemental concentrations, displayed in the biplot of two PC, the importance of variables (in descending order) in PC1 was as follows: Fe > Cd > Na > Cr > Al > Mg > Mn > K > Ni > Pb > Zn > Ca > P > N > Cu. The order of importance of variables in PC2 was different: N > P > Cu > Pb > Ni > Mg > Mn > Al > K > Ca > Zn > Cd > Fe > Cr > Na.

## 3. Discussion

For a long time, *J. communis* ionomic parameters have received significant attention in various regions of Europe [8,33,40,41,44,46,47]. The deficiency, normal, or excess/toxic concentrations of elements for the growth of conifers were determined at the end of the last century [48,49,50,51,52,53,54,55].

In Lithuania, the ionome of *Pinus sylvestris* and *Picea abies* were examined near local industrial pollution sources [55,56]. The only assessment of *Juniperus communis* ionome was performed two decades earlier, regardless of the needles’ age [40].

### 3.1. Macroelements

In conifer needles, a concentration of N lower than 12,000–14,000 μg/g d. m. is considered a nitrogen deficiency. Normal or ecological optimum values range between 14,000 and 18,000 μg/g d. m. Ecological excess or physiological optimum values are from 18,000 to 23,500 μg g^–1^ d. m., and physiological excess values that affect and inhibit the growth of the plant are from 23,500 to 35,000 μg/g d. m. [48,50,51,52]. In our study of *J. communis,* the mean concentration of N in current-year needles (Figure 1) was 15,422 μg/g d. m. The mean concentration of N in same age needles of *J. communis* in Italy was much lower (8450 μg/g d. m.) [46]. The concentration of N in current-year needles of Scot pine in Lithuania (near industrial pollution sources) ranged from 12,300 to 20,030 μg/g d. m. [56]. The concentration of Ca in conifer needles lower than 400 to 2000 μg/g d. m. is considered a deficiency [48,55]. The mean concentration of Ca in *J. communis* in Lithuania was 10,352 μg/g d. m. and in Italy it was 12,300 μg/g d. m. [46]. The concentration of Ca in Scot pine needles in Lithuania ranged between 6100 and 10,400 μg/g d. m. [56]. In conifer needles, a concentration of P lower than 1300 μg g^–1^ d. m. is considered a phosphorus deficiency [48]. In our study, the mean concentration of P in *J. communis* was 1516 μg/g d. m.; this was similar to the concentration of P in *Pinus sylvestris* in Lithuania (980 to 1480 μg/g d. m.) [56] and Scandinavian countries (819 to 2120 μg/g d. m) [48]. In Italian *Juniperus communis*, the mean concentration of P was significantly higher (6720 μg/g d. m.) [46]. In conifer needles, a concentration of K lower than 4000 μg/g d. m. is considered a potassium deficiency [48]. The mean concentration of K in *J. communis* in Lithuania was 3145 μg/g d. m. and the mean concentration of K in *J. communis* in Italy was 3500 μg/g d. m. [46]. In Lithuanian *Pinus sylvestris,* the concentration of K ranged from 450 to 7650 μg/g d. m. [56]. In conifer needles, a concentration of lower than 250 to 400 μg/g d. m. is considered a major magnesium deficiency (visible symptoms can be detected) [57]. If the concentration is within 400 to 600 μg/g d. m., this is considered a minor deficiency (growth retardation might be observed). Meanwhile, normal values range between 600 to 900 μg/g d. m. and optimal values are 800 to 1300 μg/g d. m. [46,47,48]. In our study, the mean concentration of Mg in *J. communis* was 608 μg/g d. m. The mean concentration of Mg in *J. communis* in Italy was three times higher (1800 μg/g d. m.) [38]; in Lithuanian *P. sylvestris*, the concentration of Mg ranged from 1200 to 1710 μg/g d. m. [56].

### 3.2. Microelements

In conifer needles, a concentration of Mn lower than 10 to 20 μg/g d. m. is considered a manganese deficiency [48]. In our study, the mean concentration of Mn in current-year needles of *J. communis* (Figure 1) was 275.4 μg/g d. m. The mean concentration of Mn in *J. communis* in Italy was much lower (85 μg/g d. m.) [46]. In contrast, the study conducted in Lithuania 20 years earlier reported the concentrations of Mn between the range of 177 to 2930 μg/g d. m. for *J. communis* and between the range of 33 to 3500 μg/g d. m. for *Picea abies* [40]. In conifer needles, a concentration of Fe higher than 500 μg/g d. m. is considered as ecological excess or physiological optimum values [48]. In our study, the mean concentration of Fe in *J. communis* was 65.5 μg/g d. m.; this was very similar to Italian data (54 μg/g d. m.) [46]. Iron concentration in Lithuanian *Pinus sylvestris* was in the range similar to that found in our study (44 to 94 μg/g d. m.) [56]. In conifer needles, a concentration of Zn lower than 8 to 15 μg/g d. m. is considered a deficiency in zinc; concentrations of 300 to 600 μg/g d. m. are poisonous to the plant [48]. In our study, the mean concentration of Zn in *J. communis* was 16.55 μg/g d. m. The concentration of Zn in the previous study (24 sites in Lithuania) ranged from 16 to 30 μg/g d. m [40], while the mean concentration of Zn in *J. communis* in Italy was 23 μg/g d. m. [46]. In the current-year–four-year needles of *Picea abies* (48 sites in Lithuania) the concentration of Zn ranged from 10 to 72 μg/g d. m) [40], and the concentration of Zn in Lithuanian *Pinus sylvestris* was within the range of 42.9 to 75.3 μg/g d. m. [56]. In conifer needles, a concentration of Cu lower than 2 to 3 μg/g d. m. is considered a copper deficiency. Normal or ecological optimal values range between 5 and 30 μg/g d. m.; ecological excess or physiological optimum values range between 20 and 30 μg/g d. m., while concentrations between 20 and 100 μg/g d. m. are poisonous to the plant [48]. The mean concentration of Cu in *J. communis* in our study was 4.43 μg/g d. m., nearly the same as the mean concentration of Cu in *J. communis* in Italy (4 μg/g d. m.) [46] and within the range of concentrations of Cu in *Pinus sylvestris* in Lithuania (3.62 to 5.79 μg/g d. m.) [56]. Concentrations of K, Ca, Fe, Zn, Al, Cu, Ni, and Cd in our *J. communis* populations were very similar to Italian *J. communis*, growing quite distinct climates and edaphic conditions. This shows that nutrient concentrations are tightly related to genome. Such evidence further supports the validity of the Ellenberg indicatory value for soil richness along a wide geographical scale.

### 3.3. Non-Essential Elements

According to our study, the mean concentration of Al in the needles of *J. communis* was 25.26 μg/g d. m (Figure 1). The mean concentration of Al in *J. communis* in Italy was 30 μg/g d. m. [46] and concentrations of Al in Lithuanian *Pinus sylvestris* ranged from 25 to 190 μg/g d. m. [56]. In conifer needles, a concentration of Ni between 0.5 to 5 μg/g d. m. is considered as normal or ecologically optimal values, whereas concentrations of 10 to 100 μg/g d. m. are poisonous to the plant [39,58]. In our study, the mean concentration of Ni in *J. communis* was 0.73 μg/g d. m. The mean concentration of Ni in *J. communis* in Italy was 1 μg/g d. m. [46]. The concentrations of Ni in Lithuanian *Pinus sylvestris* ranged from 0.58 to 2.21 μg/g d. m. [56]. The mean concentration of Pb in *J. communis* in our study was 0.265 μg/g d. m. The concentrations of Pb in *J. communis* ranged from 0.19 to 1.23 μg/g s. m in the previous study in Lithuania [40]. The mean concentration of Pb in *J. communis* in Italy was several times higher (1 μg/g d. m.) when compared to our results [46]. The mean concentration of Pb in the current-year–four-year needles of *P. abies* in Lithuania ranged from 0.32 to 1.4 μg/g d. m. [40]; concentrations of Pb in Lithuanian *Pinus sylvestris* were within the range of 0.66 to 2.01 μg/g d. m. [56]. Our findings of low Pb concentrations in the needles of juniper corresponded to the background concentrations of this element found in the leaves of deciduous trees (*Salix* spp., *Populus tremula* L., *Frangula alnus* Mill., *Quercus robur* L., *Tilia cordata* Mill., *Acer platanoides* L., *Alnus* spp.), and herbaceous plants (*Agrostis* spp., *Achillea millefolium* L.) sampled in various sites in Lithuania [59]. In our study, the mean concentration of Cr in *J. communis* was 0.305 μg/g d. m. The concentration of Cr in *J. communis* in the previous Lithuanian study ranged between 0.07 and 0.39 μg/g s. m [40], the mean concentration of Cr in *J. communis* in Italy was 2 μg/g d. m. [46], and the concentration of Cr in the current-year–four-year needles in Norway spruce in Lithuania ranged from 0.12 to 0.45 μg/g d. m. [40]. The concentration of Fe and Ni in *J. communis* needles in Scotland (Fe 11.7–310 μg/g d. m.; Ni 6.9–32 μg/g d. m.) was similar to our results, but the concentration of Cr was higher in Scotland (Cr 9.7–15 μg/g d. m.) [58]. The mean concentration of Cd in *J. communis* in our study was 0.034 μg/g d. m. In the previous Lithuanian study, the concentrations of Cd in *J. communis* ranged from 0.03 to 0.23 μg/g d. m. [40], the mean concentration of Cd in *J. communis* in Italy was 0.01 µg/g d. m. [46], and the mean concentration of Cd in the current-year–four-year needles in *Picea abies* in Lithuania ranged from 0.01 to 0.29 µg/g d. m. [40].

### 3.4. Macro-, Micro-, Non-Essential Element Concentration Relationship to Habitat Type 

Concentrations of nutrient elements in *Juniperus thurifera* needles varied widely and depended on habitat [60]. When compared to other habitats, *J. communis* growing in coastal brown dunes covered with natural Scots pine forests (B) had high needle concentrations of Fe, Zn, Ni, Na, Cr, Pb, and Cd, moderate concentrations of N, K, Ca, and Cu, and low concentrations of Mg and Al (Figure 2). The coastal brown dunes covered with natural Scots pine forests (B) are dominated by sandy soils covered by a thin layer of organic matter that is affected by intense wind erosion. Analysis of elements in *J. communis* needles showed that the concentration of Mg (591 µg/g d. m.) was significantly lower in coastal brown dunes covered with natural Scots pine forests (B) when compared to other habitats. The Mg concentration of 400 to 600 μg/g d. m. is considered as a mild deficiency [53,54]. Significantly higher concentrations of Na (9.49 µg/g d. m.) and Cr (0.531 µg/g d. m.) were also determined in the Scots pine forests habitat (B) when compared to other habitats (*Juniperus communis* scrubs (F), transition mires and quaking bogs (D), xero-thermophile fringes (E), and subcontinental moss Scots pine forests (G)). High concentrations of Na are typical for acidic or salty wetlands [61]. The high concentration Na found in population 1 B could be related to its proximity to the sea. Increased Na^+^ ions may replace K^+^ ions [62] and affect the plant’s activity. The highest concentration of Cd was 0.105 µg/g d. m. In our study, concentrations of heavy metals (Cd, Cr, and Ni) in the needles of *J. communis* did not reach levels that are harmful for plants.

When compared to other habitats, the needles of *J. communis* growing in *Juniperus communis* scrubs (F) had high concentrations of K, moderate concentrations of Mn, and low concentrations of Zn and Cu. *Juniperus communis* scrubs (F) are distinguished by a high density of *J. communis* (1747 units/ha) [63]. Only the concentration of Zn (13.05 µg/g d. m.) was significantly lowest in *Juniperus communis* scrubs (F), within a range indicating a deficiency in conifer needles (8 to 15 μg/g d. m.) [64]. Zinc deficiency results in a stronger response when compared to the deficiency of other microelements [65]. Zinc influences the production of auxin, and when the production of this hormone is disrupted, plant growth might be limited [66,67].

According to studies in various European countries, ecological optimum of N in conifer needles is 14,000 to 18,000 μg/g d. m. and N deficiency is assumed in case its concentration is lower than 12,000 to 14,000 μg/g d. m. [48,68]. *Juniperus communis* needles of the same age sampled in the mixed oak–pine forest *Querco*–*Pinetum* (Poland) had very similar N concentration as in some habitats in our study (i.e., coastal brown dunes covered with natural Scots pine forests (B), *Juniperus communis* scrubs (F), and transition mires and quaking bogs (D)) and lower concentration than in the rest of our study habitats (i.e., subcontinental moss Scots pine forests (G) and xero-thermophile fringes (E)) [33].

Compared to all other habitats (B, F, G, and E), our populations in transition mires and quaking bogs (D) had significantly lower concentrations of the main nutritional elements N (12,176 µg/g d. m.), P (1054 µg/g d. m.), and K (2916 µg/g d. m.). Only the concentration of P in the populations in transition mires and quaking bogs (D) did not differ from the F habitat. When compared to other habitats, the mean concentrations in the needles in transition mires and quaking bogs (D) were highest for Ca, moderate for Fe, Zn, Al, and Pb, and lowest for N, P, K, Ni, Cr, and Cd. The concentrations of needle calcium causing a normal growth are 3000 to 12,800 μg/g d. m. and its deficiency falls into the interval of 400 to 3000 μg/g. m. [53]. In our study, needle deficiency in P (<1300 μg/g d. m. [48] was found in *J. communis* growing in transition mires and quaking bogs (D) and *Juniperus communis* scrubs (F). The highest concentration of Ca (14,723 µg/g d. m.) was found in population 8 D in the lakeside area, which is dominated by calcareous soil. Compared to other habitats, *J. communis* growing in transition mires and quaking bogs (D) had the lowest concentration of Ni (0.21 µg/g d. m.). Copper concentrations within a range of 5 to 30 μg/g d. m. are assumed as normal, whereas concentrations within 2 to 3 μg/g d. m. are deficient for conifer needles [48]. In our study, the lowest Cu concentrations (ranging from 1.54 to 1.89 µg/g d. m. and falling into the interval of deficiency) were determined in *Juniperus communis* scrubs (F) and transition mires and quaking bogs (D). Compared to other habitat types, transition mires and quaking bogs (D) and xero-thermophile fringes (E) had the lowest concentrations of Mn (ranging from 56.7 to 42.2 μg/g d. m.) that were still within the interval of concentrations required for the normal growth (20 to 300 μg/g d. m.) [69]. Fe chlorosis often occurs when the concentration of Fe is decreased [70] or if the concentration of Mn exceeds that of Fe by twice or more [71]. Thus, Fe should always be greater than Mn in the environment to avoid the blockage of Fe. In our sites, the situation is not favorable in respect of the Fe and Mn ratio due to the low range of Fe concentrations (20.2 to 115 μg/g d. m.) and the high range of Mn concentrations (30.2 to 939.2 μg/g d. m.). Elevated Cu concentration can also lead to Fe deficiency [62], but this was not the case in our study as Cu concentrations in *J. communis* were within the range of either deficiency (1.10 to 7.46 μg/g d. m.) or normal growth (2 to 3 μg/g d. m.) [48].

Compared to other habitats, the concentration of elements in the needles of *J. communis* in subcontinental moss Scots pine forests (G) were high for N, P, Mg, and Mn, moderate for K, Zn, and Cd, and low for Fe, Na, Cr, and Pb. Subcontinental moss Scots pine forests (G) are distinguished by having optimal conditions for *J. communis*, which forms forest undergrowth below the crown of Scots pine. In this habitat, significantly higher concentrations of K (3266 µg/g d. m.) and P (1765 µg/g d. m.) were determined when compared to other habitats. The lowest concentration of Pb (0.186 µg/g d. m.) was found in this habitat, although it did not differ significantly from the xero-thermophile fringes (E). The concentration of Pb did not fall between the interval of concentrations known as toxic (5 to 10 μg/g d. m.) [54]. The comparison of elemental concentrations in the needles of *J. communis* between xero-thermophile fringes (E) and other habitats revealed that in xero-thermophile fringes the concentrations of Mg, Cu, and Al were high, concentrations of P, Ni, Na, and Cr were moderate, and concentrations of Mn and Fe were low. The highest Mg concentrations (613 µg/g d. m.) were found in both subcontinental moss Scots pine forests (G) and xero-thermophile fringes (E), representing the levels required for normal growth.

In most cases, needle concentrations of N, P, K, and Mg were positively interrelated, but negatively related to Ca (Figure 3). Positive correlations were found between needle concentrations of Pb, Cr, and Ni. In some cases, Zn and Cu positively correlated with macroelements. Increased concentrations of Al corresponded to increased concentrations of Cu and Zn and to decreased concentrations of Al, Fe, Mn, Zn, Pb, Cr, and Cd. In case of significant correlations between Pb, Cr, and Cd, the higher concentration of one heavy metal corresponded to the higher concentration of the other.

Discriminating between the populations (Figure 4), the most important variables for the PC1 were concentrations of Cd, Al, Cr, and Fe and the most important for the PC2 were concentrations of P, N, Mg, Ca, Cu, and K. Discriminating between the habitats (Figure 5), important variables for the PC1 were concentrations of Fe and Cd, while concentrations of N and P were important for the PC2. Principal component analyses using concentrations of 15 elements as variables for the discrimination between populations or habitats allowed authors to distinguishing the F and B habitats from the E habitat (PC1) and F and D habitats from the G habitat (PC2).

In parallel to ionomic studies of *J. communis*, genetic diversity at inter-simple sequence repeat (ISSR) loci was analyzed [39] and coverage of *J. communis* was estimated. Molecular diversity (percentage of polymorphic ISSR loci) of *J. communis* was lowest for transition mires and quaking bogs (D, 42.9 % as a mean) and highest for *Juniperus communis* scrubs (F, 68.2 %). Molecular diversity was intermediate for subcontinental moss Scots pine forests (G, 43.9 % as a mean), for coastal brown dunes covered with natural Scots pine forests (B, 48.0 %), and for xero-thermophile fringes (E, 48.5 %) [72]. Coverage by *J. communis* also depended on the habitat type, the smallest being for transition mires and quaking bogs (D, 5 % as a mean) and the highest for *Juniperus communis* scrubs (F, 60 %). It was intermediate for subcontinental moss Scots pine forests (G, 13% as a mean), for coastal brown dunes covered with natural Scots pine forests (B, 15 %), and for xero-thermophile fringes (E, 36 %) [72]. As such, the lowest polymorphism at ISSR loci and the lowest coverage by *J. communis* in transition mires and quaking bogs corresponded the worst nutritional status in respect to needle concentrations of N, P, Mg and Cu. The highest polymorphism and the highest coverage by *J. communis* in *Juniperus communis* scrubs (F) corresponded to the highest needle concentration of K and deficiency in P, Mg, Zn, and Cu.

For various plant species, including junipers, Ellenberg indicator values (EIV) of neighboring herbaceous plant species are widely used to evaluate environment in the sites without the need to perform physico-chemical measurements [73,74,75,76,77]. During evaluation of *J. communis* environments by EIV, the soil richness in nutrients (EIV-N) [35] showed low fertility for all habitats; it was lower than the middle value (5) and ranged in the interval of 2.7 to 4.4. Soil reaction (EIV-R) ranged in the wider interval of 2.7 to 7.4 [72]. Our soil acidity values corresponded well with the findings of chemical analyses performed by other researchers [78]. According to pH, the most calcareous soil (EIV-R—7.4) was in xero-thermophile fringes (E) where juniper needles had lowest concentrations of Mn and Fe and intermediate values of polymorphic DNA and coverage.

*Juniperus communis* belongs to the list of plant taxa most frequently mentioned in the records of Lithuanian, Latvian, and Estonian folk medicine. Juniper was grown as a houseplant and used to treat respiratory and skin diseases. It was also applied as Sauna whisk. Juniper tea was used against bad breath, headache, hypersalivation, abdominal pain, chest pain, scabies, bacterial infection, tuberculosis, etc. [34,79,80,81]. This species is included into Estonian, Latvian, and Lithuanian national pharmacopoeias [82]. In order to clarify the best conditions for collecting medicinal raw material of juniper depending on the condition of the plant and environmental factors, intensive qualitative and quantitative analysis of essential oils has been conducted in Lithuania and other Baltic countries [22,23,78,79,83,84]. Our elemental analysis revealed that the ionome of juniper growing in distinct habitats is different, therefore junipers collected in a single habitat would be the most suitable for a homogeneous medicinal raw material. According to the nutritional status of the needles, junipers growing in the subcontinental moss Scots pine forests (G) would be the most suitable for this purpose.

## 4. Materials and Methods

### 4.1. Study Sites and Sampling Material

**Figure 6:**
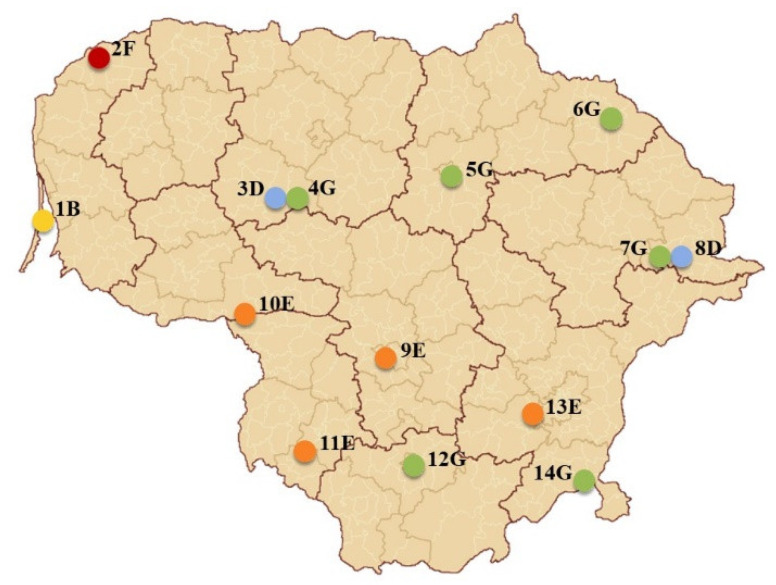
Sampling sites with common juniper (*Juniperus communis*) (Lithuania). 1–14—sites; habitat types: B—coastal brown dunes covered with natural Scots pine forests, F—*Juniperus communis* scrubs, D—transition mires and quaking bogs, G—subcontinental moss Scots pine forests, E—xero-thermophile fringes.

For the study, 14 sites of *J. communis* representing five different habitats and covering most of Lithuania’s territory was selected (Figure 6). Following classification by Davies et al. (2004), habitat types were named as follows: (1) type B1.71 (B)—coastal brown dunes covered with natural Scots pine forests; (2) type F3.16 (F)—*Juniperus communis* scrubs; (3) type D2.3 (D)—transition mires and quaking bogs; (4) type G3.42111 (G)—subcontinental moss Scots pine forests; (5) type E5.21 (E)—xero-thermophile fringes. Site names were abbreviated according to location (the number) and habitat type (the first letter of the habitat code: B, F, D, G, and E).

Geographic distances between sites ranged from 46 to 339 km. Two pairs of neighboring sites (3 D and 4 G, and 7 G and 8 D) located within 0.5 km distance of each other were included as they differed in habitat type. Geographical ranges between the study sites covered 56°07′ to 54°11′ latitude, 21°06′ to 26°30′ longitude and 25 to 187 m altitude [39].

Sampling was carried out during a 2-day period within the 2nd week of September 2013. At each site, shoots with current-year needles were harvested from the middle part of the crown. If a tree or a bush was higher than 2.5 m then shoots were taken at the height of 1.75 to 2.00 m. The middle of September was chosen for sampling because current-year needles finish their growth at this time. Current-year needles of *J. communis* were examined in many other ionomic studies [33,46,47]. Needle loss starts from the second or third year and differs depending on the site; therefore, mature current-year needles of *J. communis* are most suitable for elemental analysis. Because of the varying opinions regarding the sexual dimorphism of and differences in the chemical composition between female and male individuals [33,44,47,85], samples were taken only from female plants to ensure that plant material was as homogenous as possible. Moreover, female seed cones are used for medical and economical purposes [30,31,79,86]. At each site, three independent batches of needles were collected from the same individuals used for molecular studies [39]. Shoots were placed in separate paper bags and dried at room temperature, then placed in a thermostat at 30 °C until a constant mass was attained. Any small fragments of the bark or buds of the previous year’s shoots were carefully eliminated. Needles were milled into fine powder using a Retsch MM400 tungsten carbide grinder (Germany).

### 4.2. Analysis of Nitrogen and Macro-, Micro- and Non-Essential Elements

The Kjeldahl method [87] was used to determine nitrogen (N) in the current-year needles of *J. communis*. Milled samples were digested in a Semi-Automatic Digester Instrument, model DK-20S (VELP Scientifica, Italy). Ammonia distillation using the Kjeldahl standard method was performed in an Automatic Distillation and Titration System, model UDK 159 (VELP Scientifica, Italy) as described earlier [88]. Nitrogen concentration was measured as micrograms per gram in a dry mass (μg/g d. m.). The quality assurance of analysis was ensured using standard reference materials NIST 1515 and NIST 1575 and a certificated reference material CRM 125045.

To determine macro-, micro-, and non-essential elements in current-year needles of *J. communis,* milled samples were digested in an ETHOS One microwave digestion system with a high pressure segmented rotor SK10 (Milestone, Italy). Digested samples were analysed by atomic absorption spectrophotometer, model AA–6800, controlled by WizAArd 2.31 (Shimadzu, Japan) software. Calibration lines for each element were constructed using standard solutions (1000 μg/mL, Sharlau, Spain). Standard reference materials NIST 1575 and NIST 1547 were used for calibration quality assurance.

Phosphorus concentration of mineralized *J. communis* samples was determined spectrophotometrically [89]. The colorimetric analysis was carried out using an acidic molybdate solution and ascorbic acid. Measurements were performed at a wavelength of 627 nm with a dual-beam spectrophotometer UV-1800 (Shimadzu, Japan).

### 4.3. Comparison of Ionomic Data with Environmental Parameters

To relate ionomic data with other characteristics of *J. communis*, the genetic diversity of juniper populations was evaluated using ISSR markers [39]. For comparison of ionomic data with environmental parameters, undergrowth trees and shrubs, herbaceous species, and dwarf shrubs were recorded at each site following Braun-Blanquet [90] and the abundance of each species was evaluated using EIV [35] as described in detail previously [72,75,76].

### 4.4. Statistical Analysis

To compare different populations and population groups representing different habitats, descriptive statistics were calculated using R software package (v. 4.2.2) and R package “DescTools” (v. 0.99.42) [91,92]. To compare macro-, micro-, and non-essential elements concentrations between population groups, the Kruskal–Wallis test was used [93]. To relate macro-, micro-, and non-essential elements, Pearson rank correlations were calculated and a correlogram was constructed using R package “corrplot” (v. 090) [94]. Principal component analysis was carried out using macro-, micro-, and non-essential elements as variables using R software [92].

## 5. Conclusions

1. Of all elements analyzed in current year needles, the concentrations of N and P were the most important for the discrimination between *J. communis* habitats.

2. The worst nutritional status according to the concentrations of N, P, Mg, and Cu in the needles of *J. communis* in transition mires and quaking bogs corresponded to the lowest polymorphism at ISSR loci and the lowest coverage of this species.

3. The highest needle concentration of K and needle deficiency in P, Mg, Zn, and Cu in the habitat of *Juniperus communis* scrubs corresponded to the highest polymorphism at ISSR loci and the highest coverage.

4. Despite large differences between the sites, the concentrations of Cd, Cr, and Ni in the needles of *J. communis* did not reach levels harmful to plants.

For evaluation and preservation of diversity of *J. communis*, ionomic assessment should be included and management strategies should be directed to retain variety of habitats, encompassing both widely spread and less common ones.

## Data Availability

Not applicable.
